# Clinical Significance of Serum-Derived Exosomal LINC00917 in Patients With Non-Small Cell Lung Cancer

**DOI:** 10.3389/fgene.2021.728763

**Published:** 2021-12-24

**Authors:** Dani Xiong, Chuanlin Wang, Zhaohui Yang, Fusen Han, Huaibing Zhan

**Affiliations:** Department of Respiratory Disease, Qingdao Huangdao District Central Hospital, Qingdao, China

**Keywords:** lung cancer, NSCLC, exosome, biomarker, lncRNA

## Abstract

**Background:** In this study, we aimed to explore the diagnostic potential of serum-based exosomal long intergenic noncoding RNA 917 (LINC00917) in non-small cell lung cancer (NSCLC).

**Methods:** Exosomes were extracted from NSCLC patients’ serum samples. Exosomal LINC00917 expression levels were compared, by qRT-PCR, between cancer patients and healthy controls, as well as sub-populations of cancer patients. The association between exosomal LINC00917 expression and NSCLC patients’ clinicopathologic factors were investigated, and receiver operating characteristic (ROC) curves were drawn. In addition, NSCLC patients’ overall survivals (OSs) was examined based on exosomal LINC00917 expression and further evaluated by the cox regression analysis.

**Results:** Serum-derived exosomal LINC00917 was highly expressed in NSCLC patients, and further upregulated in stage III/IV cancer patients. Exosomal LINC00917 yielded modestly good under the curve (AUC) values. Upregulated exosomal LINC00917 expression was closely associated with cancer patients’ advanced stages and shorter OSs.

**Conclusion:** Serum-derived exosomal LINC00917 may hold diagnostic potential for patients with non-small cell lung cancer.

## Introduction

Lung cancer is the leading cause of cancer death among male patients and second leading cause of mortality among female patients in the world (Sung et al., 2021). In the United States of America, although the total cases of lung cancer declined from 2018 to 2020, lung cancer still caused more deaths than breast, prostate, colorectal, and brain cancers combined (Siegel et al., 2020). In China, lung cancer is poised as a heavy burden to public health as it ranks the first in male cancer incidence spectrum (24.17%) and the second in females (15.02%) (He et al., 2020). The most common lung cancer is non-small cell lung cancer (NSCLC), which accounts for ∼85% of total lung cancer cases and has diverse pathological features (Chen et al., 2014). It is critical to find suitable biomarkers for NSLCLC, so cancer patients can be diagnosed and treated efficiently.

Exosomes are membrane-bound extracellular vesicles that contain secreted proteins, DNAs or RNAs, and then taken up by adjacent cells to affect their functions (Butler, 2002; Peterson et al., 2015). Emerging evidence has indicated that exosomes regulated intercellular communications among cancer cells and their adjacent microenvironments, thus contributing to various aspects of cancer development (Li et al., 2017; Mashouri et al., 2019; Zhang and Yu, 2019). Specifically, exosomes are often expressed in human body fluids, such as peripheral bloods, and their constituted protein/DNA/RNA expression levels were found to be closely associated with cancer cell malignancies, thus making them potential circulating biomarkers for human cancers (Jia et al., 2017; Tomasetti et al., 2017; Kok and Yu, 2020; LeBleu and Kalluri, 2020). Thus, it is important to exploit the potential candidates of exosomal biomarkers in clinical application of NSCLC.

Long non-coding RNAs (lncRNAs) are a group of non-protein-coding transcripts that are longer than 200 nucleotides and had been recently identified to play important roles in regulating human cell development and functions (Chen and Yan, 2013; Jarroux et al., 2017; Kazimierczyk et al., 2020). Emerging evidence has demonstrated that lncRNAs can be aberrantly expressed in human cancer cells and play critical roles in regulating tumor cell carcinogenesis and development (Yang et al., 2014; Bhan et al., 2017; Peng et al., 2017). In addition, strong evidence has shown that human tumor cell-derived lncRNAs can be packaged into exosomes and then transported to peripheral circulating system, thus making exosomal lncRNAs potential candidates of noninvasive cancer biomarkers (Zhao et al., 2018; Chen et al., 2019; Kelemen et al., 2019).

Among many of the human-disease-associated lncRNAs, long intergenic noncoding RNA 917 (LINC00917) was initially identified to contribute to pathogenesis of intervertebral disc degeneration (Chen et al., 2015), and associate with Alzheimer’s disease (AD) (Deming et al., 2016). Recently, LINC00917 was found to be aberrantly expressed in human colorectal tumors (Fattahi et al., 2019). However, its association with lung cancer, or other human cancers, has never been elucidated.

In this study, we examined LINC00917 expression levels in serum exosomes of NSCLC patients, and then evaluated the correlations between exosomal LINC00917 and clinicopathologic factors of NSCLC. In addition, we used receiver operating characteristic (ROC) curve analysis and overall survival (OS) analysis to estimate the diagnostic potential of exosomal LINC00917. Our results indicated that serum-derived exosomal LINC00917 had significantly clinical implications as a non-invasive prognositc factor for NSCLC.

## Materials and Methods

### Ethics Statement

All clinical and experimental procedures were reviewed and approved by the Human Study Ethics Committees at the Qingdao Huangdao District Central Hospital in Qingdao City, Shandong Province, China. Informal consent forms were signed by all participating patients prior to the onset of the study. All protocols were performed in accordance with the Declaration of Helsinki (World Medical Association, 2014).

### NSCLC Patients

A total of 179 patients diagnosed with non-small cell lung cancer (NSCLC) between June 2019 and April 2021 were enrolled in this study. Correspondingly, a total of 104 baseline-matched healthy control patients were selected. For all enrolled participants, clinical records were reviewed by two independent physicians, to ensure that nobody underwent major surgeries, chemotherapies radiotherapies or immunotherapies within 180 days prior to the onset of the study. In addition, NSCLC Patients’ staging was categorized in accordance with the seventh edition of AJCC staging guideline (Edge and Compton, 2010).

### Exosomal RNA Extraction and Quantitative Real-Time PCR

Venous blood samples were drawn from all participants and serum was separated using the two-step centrifugation method. Serum supernatant was processed using a Total Exosome Isolation Reagent (from serum) (Thermo Fisher Scientific, Shanghai, China) to extract exosomes. The exosomal pellets were suspended in phosphate-buffered saline (PBS, Thermo Fisher Scientific, Shanghai, China), and then processed using a Total Exosome RNA & Protein Isolation Kit (Thermo Fisher Scientific, Shanghai, China) to obtain exosomal RNA. Reverse transcription was conducted using a High-Capacity cDNA Reverse Transcription Kit (Thermo Fisher Scientific, Shanghai, China) according to the manufacturer’s recommendation. Quantitative real-time PCR (qRT-PCR) was performed using a QuantStudio™3 Real Time PCR System (Thermo Fisher Scientific, Shanghai, China). A Made-To-Order LINC00917 TaqMan Noncoding RNA assay (Thermo Fisher Scientific, Shanghai, China) was used to detect exosomal LINC00917 expression. Relative expression levels were quantified using the 2^−∆∆Ct^ algorithm.

### Statistical Analysis

All statistical analyses were conducted using a R Data Analysis App [4.0.3 GUI 1.73 Catalina build (7892)] (https://www.r-project.org/). Quantitative comparisons of exosomal LINC00917 expression levels were conducted using a Wilcoxon Ran Sum Test if there were two patient groups, whereas a Kruskal-Wallis Rank Sum Test was used if there were three or more patient groups. A pROC package (https://cran.r-project.org/web/packages/pROC/pROC.pdf) was used to draw Receiver operating characteristic (ROC) curves and calculate area under the curve (AUC). A Chi-squared test was used to analyze the significances of associating exosomal LINC00917 expression level with NSCLC patients’ clinicopathological factors. A survival package (https://cran.r-project.org/web/packages/survival/survival.pdf) was used to analyze NSCLC patients’ overall survival (OS), and a Kaplan–Meier method with log-rank was performed to compare OSs between different patient groups. Finally, univariate and multivariate cox regression models were conducted to analyze the independence of using exosomal LINC00917 level to predict NSCLC patients’ OS. For all analyses, differences with *p* values < 0.05 were termed as statistically significant.

## Results

### Exosomal LINC00917 was Upregulated in NSCLC Patients

**FIGURE 1:**
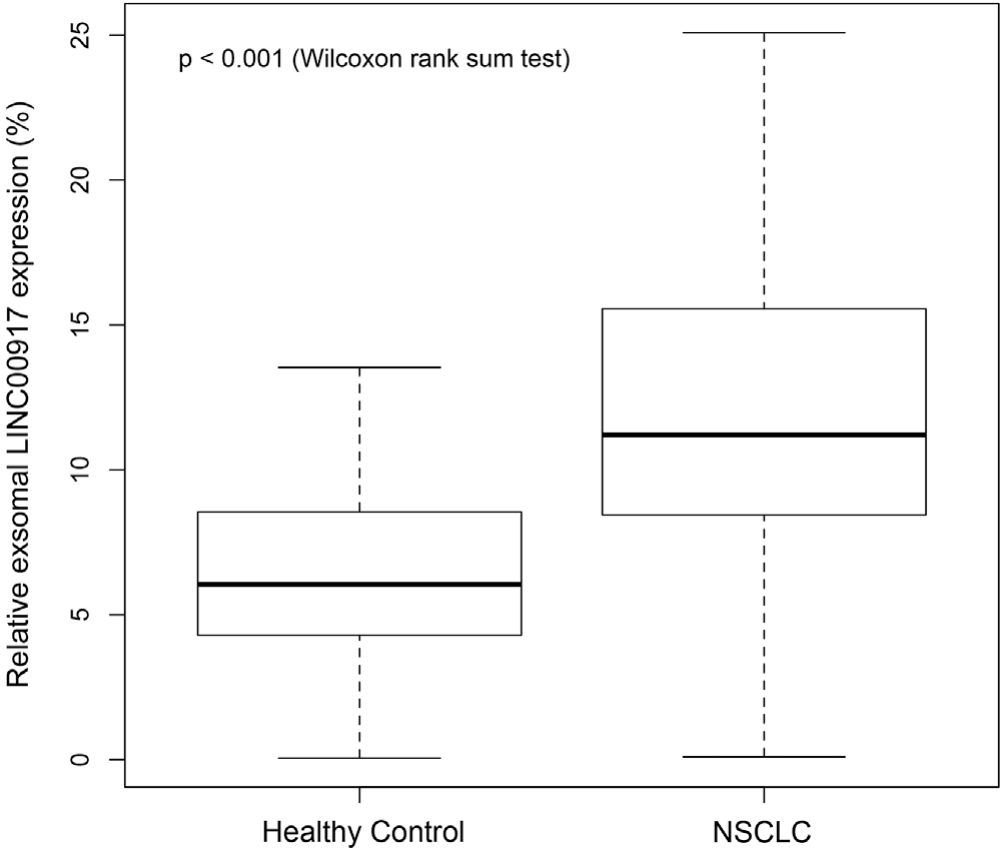
Comparison of exosomal LINC00917 between NSCLC patients and healthy controls. qRT-PCR was used to compare exosomal LINC00917 expression levels between NSCLC patients (*n* = 179) and baseline-matched healthy control patients (*n* = 104). (Wilcoxon Rank Sum Test, center lines = medians; boxes = upper and lower quartiles, vertical lines = 95% confidence intervals).

Exosomal LINC00917 expression levels were compared between NSCLC patients and healthy controls. qRT-PCR analysis showed that, exosomal LINC00917 was significantly upregulated in NSCLC patients than in healthy control patients (Figure 1, *p* < 0.001).

### Exosomal LINC00917 was Upregulated in Stage III/IV NSCLC Patients

**FIGURE 2:**
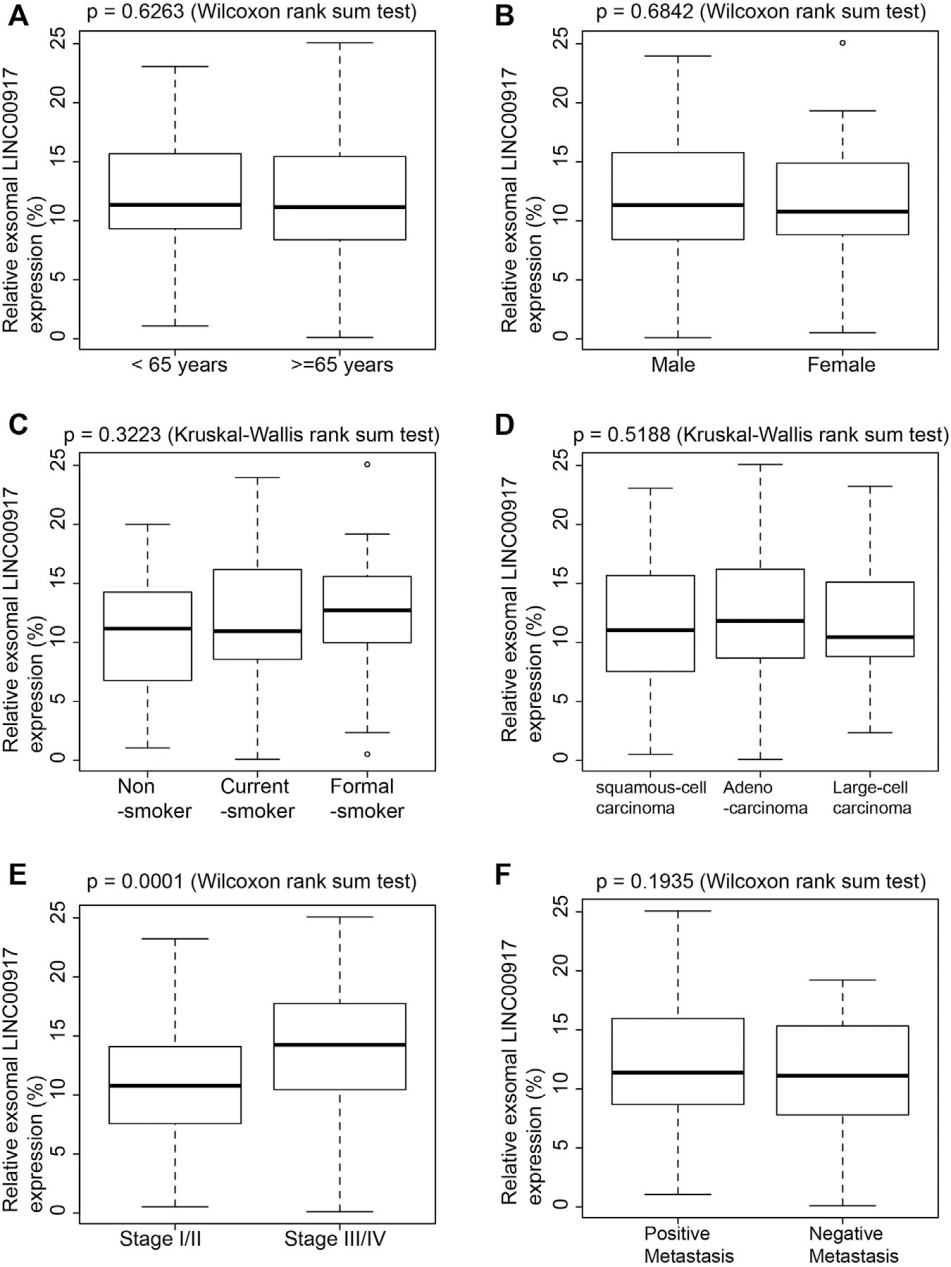
Comparisons of exosomal LINC00917 among NSCLC patient sub-populations. **(A)** exosomal LINC00917 expression levels were compared between NSCLC patients who were age 65 and older, and who were younger than 65 (Wilcoxon Rank Sum Test). **(B)** exosomal LINC00917 expression levels were compared between male and female NSCLC patients (Wilcoxon Rank Sum Test). **(C)** exosomal LINC00917 expression levels were compared among NSCLC patients who were non-smokers, current-smokers or formal-smokers (Kruskal-Wallis Rank Sum Test). **(D)** exosomal LINC00917 expression levels were compared among NSCLC patients who were diagnosed with squamous-cell-, adeno- or large-cell- carcinomas (Kruskal-Wallis Rank Sum Test). **(E)** exosomal LINC00917 expression levels were compared between Stage I/II NSCLC patients and Stage III/IV patients (Wilcoxon Rank Sum Test). **(F)** exosomal LINC00917 expression levels were compared between NSCLC patients with or without lymph-node metastasis (Wilcoxon Rank Sum Test). (In all plots, center lines = medians; boxes = upper and lower quartiles, vertical lines = 95% confidence intervals, points = outliners).

Exosomal LINC00917 expression levels were also compared among NSCLC patient sub-populations. qRT-PCR analysis showed that, exosomal LINC00917 expression levels were undifferentiated between patients aged 65 and older, and those younger (Figure 2A, *p* = 0.6263); between male and female patients (Figure 2B, *p* = 0.6842); among patients who were non-smokers, current-smokers or formal-smokers (Figure 2C, *p* = 0.3223), among patients diagnosed with squamous-cell-, adeno- or large-cell- carcinomas (Figure 2D, *p* = 0.5188), or between patients diagnosed with either positive or negative lymph-node metastasis (Figure 2F, *p* = 0.1935).

On the other hand, we discovered that exosomal LINC00917 was significantly upregulated in stage III/IV NSCLC patients than in stage I/II NSCLC patients (Figure 2E, *p* = 0.001).

### Exosomal LINC00917 was Associated With NSCLC Clinical Stages

Based on the mean value of NSCLC patients’ exosomal LINC00917 expression levels, cancer patietns were dichotomized into two sub-groups. In one group, patients (*n* = 96) had low exosomal LINC00917 expression levels. In the other group, patients (*n* = 83) had high exosomal LINC00917 expression levels. Then, the association between NSCLC patients’ clinicopathological factors and dichotomized exosomal LINC00917 expressions were analyzed using a Chi-squared test (Table 1, *p < 0.05). It demonstrated that, low or high exosomal LINC00917 distribution were undifferentiated between patients aged 65 and older, and those younger; between male and female patients; among patients who were non-smokers, current-smokers and formal-smokers, among patients diagnosed with squamous-cell-, adeno- or large-cell- carcinomas, or between patients diagnosed with either positive or negative lymph-node metastasis. However, our analysis indicated that, low or high exosomal LINC00917 distribution was significantly associated with patients’ clinical stages (I/II vs. III/IV) (Table 1, *p = 0.0055).

**TABLE 1:** Association between NSCLC patients’ clinicopathological factors and their dichotomized exosomal LINC00917 expressions (*p < 0.05).

Clinicopathological factors	Patients with low exosomal LINC00917	Patients with high exosomal LINC00917	
	Patient # (%) 96	Patient # (%) 83	*p* value
Age (years)			
<65	26 (27.08%)	23 (27.71%)	0.8971
≥65	70 (72.92%)	60 (72.29%)	—
Sex			
M	72 (75.00%)	66 (79.52%)	0.5899
F	24 (25.00%)	17 (20.48%)	—
Smoker or not			
None	33 (34.38%)	22 (26.51%)	0.2817
Current	48 (50.00%)	41 (49.40%)	—
Formal	15 (15.62%)	20 (24.09%)	—
Tumor Subtypes			
squamous-cell carcinoma	26 (27.08%)	20 (24.10%)	—
Adenocarcinoma	43 (44.79%)	43 (51.81%)	0.6422
Large-cell carcinoma	27 (28.12%)	20 (24.09%)	—
Tumor Clinical Stages			
I/II	78 (81.25%)	51 (61.45%)	0.0055*
III/IV	18 (18.75%)	32 (38.55%)	—
Tumor Lymph-node metastasis			
Positive	65 (67.71%)	61 (73.49%)	0.4956
Negative	31 (32.29%)	22 (26.51%)	—

### Exosomal LINC00917 had Good AUC Values for NSCLC

**FIGURE 3:**
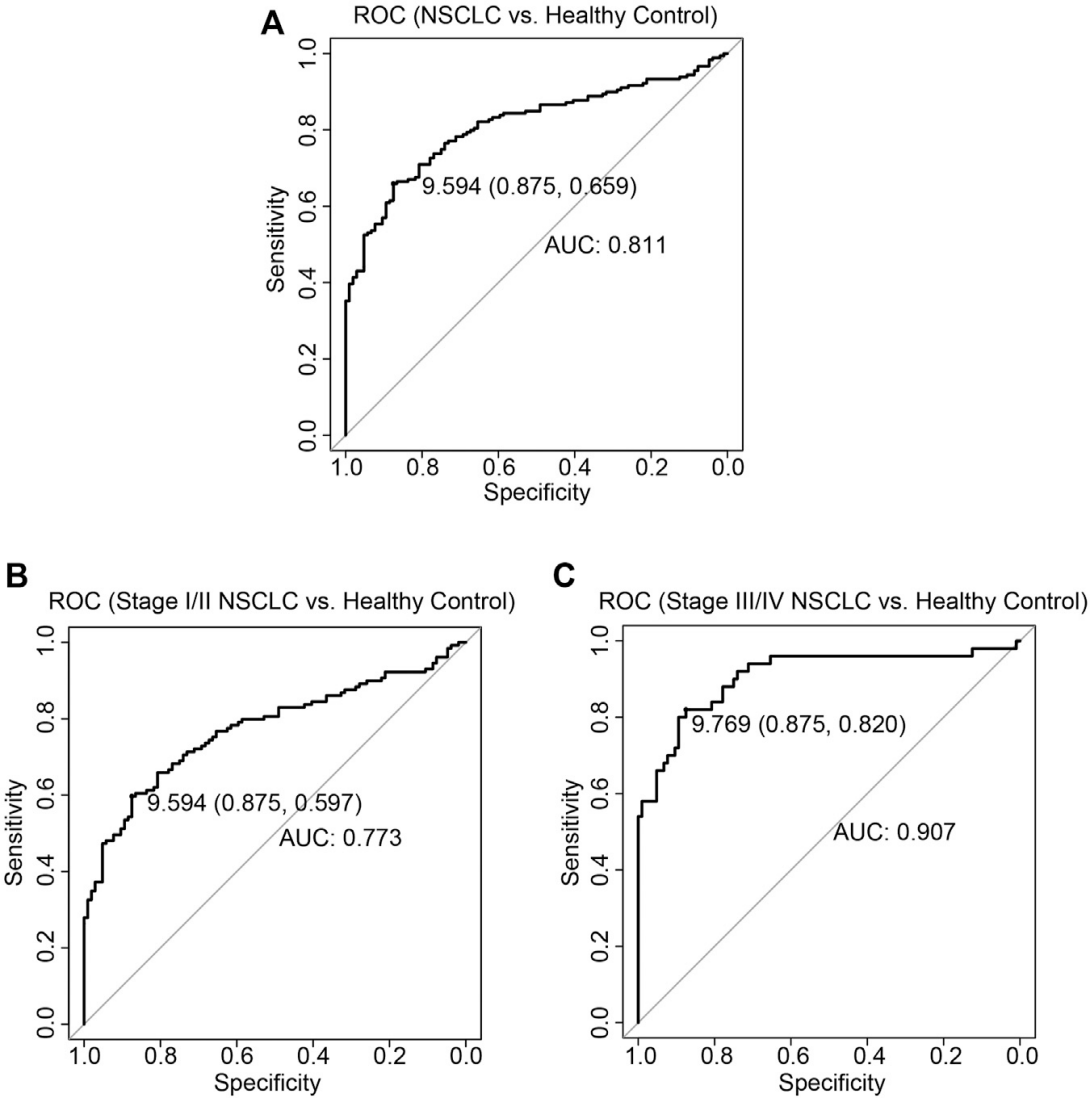
ROC for exosomal LINC00917 expression among NSCLC patient. Receiver operating characteristic (ROC) curves were drawn, and areas under the curves (AUCs) were calculated while using exosomal LINC00917 expressions to predict NSCLC **(A)**, stage I/II NSCLC **(B)**, and stage III/IV NSCLC **(C)**.

To evaluate the biomarker potential of exosomal LINC00917 for NSCLC, receiver operating characteristic (ROC) curves were drawn. It showed that. areas under the curve (AUC) were 0.811 for all NSCLC patients (Figure 3A), 0.773 for Stage I/II NSCLC patients (Figure 3B) and 0.907 for Stage III/IV NSCLC patients (Figure 3C).

### Exosomal LINC00917 was Correlated With Overall Survivals of NSCLC Patients

**FIGURE 4:**
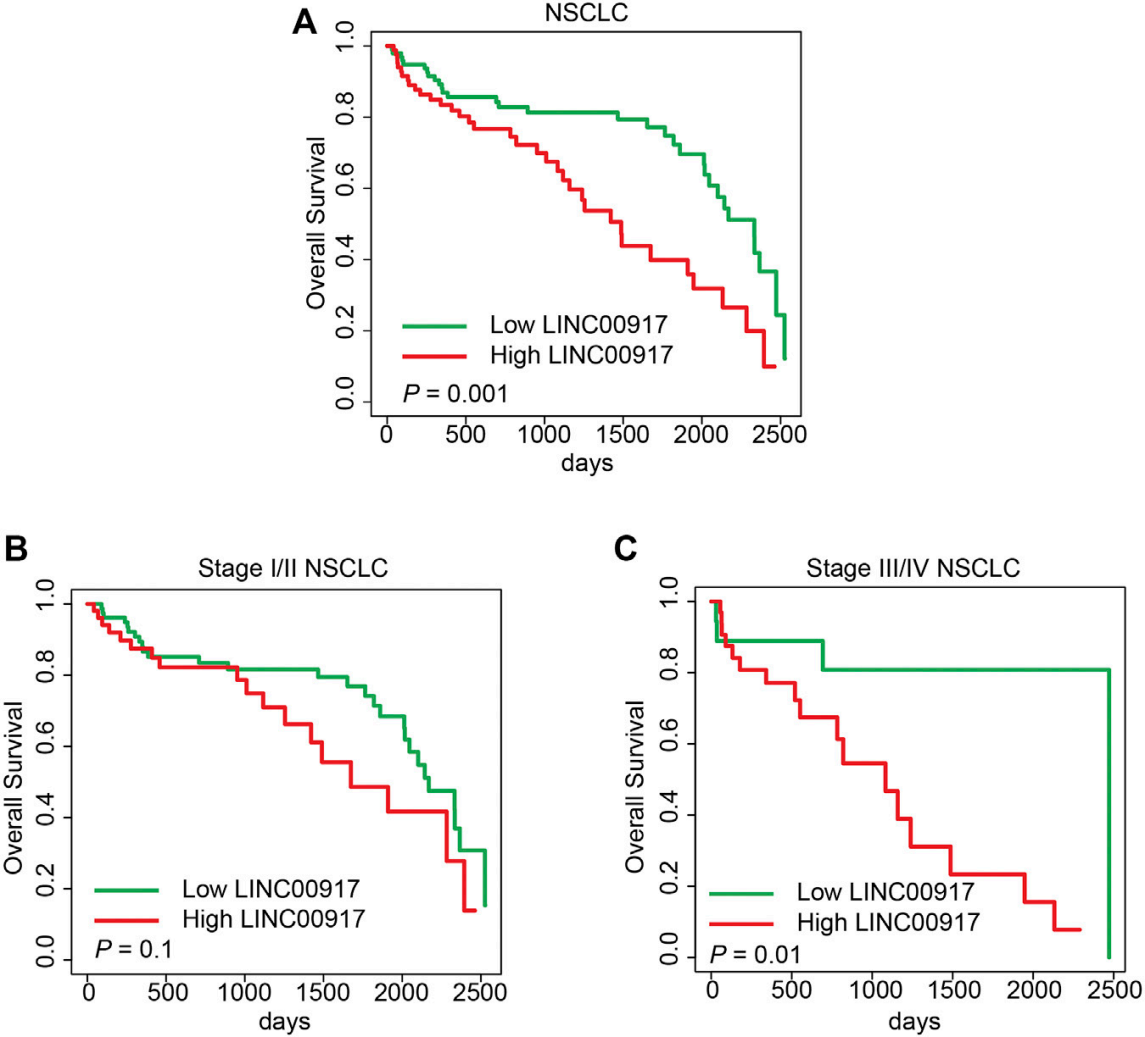
Overall survival analysis for exosomal LINC00917 expression among NSCLC patient. The overall survival (OS) curves were drawn, based on dichotomized exosomal LINC00917 expression levels, for all NSCLC patients **(A)**, stage I/II NSCLC patients **(B)**, and stage III/IV NSCLC patients **(C)**.

Finally, we looked into the correlation between exosomal LINC00917 expressions and the overall survivals (OSs) of NSCLC patients. Our data demonstrated that, high expression levels of exosomal LINC00917 were markedly correlated with shorter OSs of all participating NSCLC patients (Figure 4A, *p* = 0.001), and Stage III/IV NSCLC patients (Figure 4C, *p* = 0.01). On the other hand, exosomal LINC00917 expression level was undifferentiated for OSs of Stage I/II NSCLC patients (Figure 4B, *p* = 0.1).

The independence of using exosomal LINC00917 expression level to predict NSCLC patients’ OSs was further examined by cox regression models **(** Table 2 **)**. Based on univariate analysis, we found NSCLC patients’ OS were markedly correlated with tumor clinical stages [*p* = 0.0395, HR = 1.732, 95% CI (1.027–2.921)], tumor lymph-node metastasis [*p* = 0.0074, HR = 2.29, 95% CI (1.249–4.2)], as well as exosomal LINC00917 expression level [*p* = 0.0016, HR = 2.227, 95% CI (1.352–3.669)]. Furthermore, multivariate analysis confirmed that tumor lymph-node metastasis [*p* = 0.0025, HR = 2.547, 95% CI (1.3891–4.669)] and exosomal LINC00917 expression level [*p* = 0.0038, HR = 2.115, 95% CI (1.2732–3.514)] may act as independent factors to predict NSCLC patients’ OS.

**TABLE 2:** Univariate and multivariate cox regression analyses on the correlations between NSCLC Patients’ clinicopathological factors and overall survival (OS) (Abbreviations: HR, hazard ratio; CI, confidence interval) (*p < 0.05).

Clinicopathological factors	Comparison	Univariate analysis		Multivariate analysis	
		*p* value	HR (95%CI)	*p* value	HR (95%CI)
Age (years)	<65 vs. ≥65	0.567	0.8556 (0.5014–1.46)	—	—
Sex	Male vs. Female	0.652	0.8692 (0.4726–1.599)	—	—
Smoker or not	None vs. (Current and Formal)	0.135	1.555 (0.8713–2.776)	—	—
Tumor subtypes	Adeno- vs. (Squamous-cell and Large cell)	0.810	1.062 (0.6519–1.73)	—	—
Tumor Clinical Stages	I/II vs. III/IV	0.0395*	1.732 (1.027–2.921)	0.0073	1.634 (0.9547–2.796)
Tumor lymph-node Metastasis	Positive vs. Negative	0.0074*	2.29 (1.249–4.2)	0.0025*	2.547 (1.3891–4.669)
Exosomal LINC00917 level	Low and High	0.0016*	2.227 (1.352–3.669)	0.0038*	2.115 (1.2732–3.514)

## Discussions

Exosomal proteins or RNAs have emerged as promising non-invasive candidates for cancer prognostic factors (Jia et al., 2017; Kelemen et al., 2019; Kok and Yu, 2020; LeBleu and Kalluri, 2020). In NSCLC, a proteomic study identified urinary exosomal leucine-rich α-2-glycoprotein (LRG1) as a potential diagnosis biomarker (Li et al., 2011). A study on NSCLC patients’ serum exosomes demonstrated that high expression level of exosomal programmed cell-death ligand 1 (PD-L1) was closely associated with cancer patients’ advanced tumor stage, larger tumor size and positive metastasis (Li et al., 2019). In addition, human mature microRNA-425-3p (miR-425-3p) was found to be a potent biomarker for predicting platinum-based chemotherapy responses among NSCLC patients, and had functional role in regulating cancer cell autophagic levels (Yuwen et al., 2019). Thus, the major focus of this study was to explore serum-based exosomal RNAs in NSCLC patients, in order to identify potential non-invasive biomarkers for cancer diagnosis.

LINC00917 was a relative new epigenetic transcript, that the understandings on its expression and functional roles in human disease are very limited (Chen et al., 2015; Deming et al., 2016). Just recently, Fattahi and colleagues identified that LINC00917 was among the genetic cluster which was aberrantly upregulated in human colorectal tumors (Fattahi et al., 2019). However, the expression of LINC00917 and its potential clinical implications in other human cancers are poorly explored. Thus, in the present study, we first isolated serum exosomes and examined exosomal LINC00917 expressions in NSCLC patients. We discovered that exosomal LINC00917 was significantly upregulated in NSCLC patients’ serum samples than in healthy controls’ serum samples. The upregulation pattern of LINC00917 was in line with the discovery in human colorectal cancer (Fattahi et al., 2019). It would be interestingly to see whether LINC00917 is also upregulated in NSCLC patients’ *in situ* tumor, which would provide further evidence to identify the origin of LINC00917 upregulation among NSCLC patients.

Second in the present study, exosomal LINC00917 expression levels were compared among NSCLC sub-populations. Based on current data, we did not observe differentiated exosomal LINC00917 expressions among most of the NSCLC sub-populations. However, we did find that exosomal LINC00917 was much more upregulated in NSCLC patients with advanced tumors (Stage III/IV), than in patients with early-staged tumors (Stage I/II). Future clinical studies with expended patient pools would undoubtedly further define the correlation between exosomal LINC00917 expression and NSCLC patients’ clinicopathological factors.

Third in the present study, we discovered that exosomal LINC00917 can be a potential diagnostic biomarker of NSCLC. ROC measurement showed exosomal LINC00917 had fairly good AUC values to differentiate NSCLC patients from healthy controls. Specifically, AUC for Stage III/IV NSCLC patients was 0.907, a very good measurement as compared to other serum-based NSCLC biomarkers, such as CYFRA21-1, CA-125 or Carcinoembryonic antigen (CEA) (Cedrés et al., 2011; Grunnet and Sorensen, 2012). In addition, survival assessment supported our hypothesis, showing that high expression level of exosomal LINC00917 was significantly associated with shorter OSs among all participating NSCLC patients, as well as stage III/IV NSCLC patients. Furthermore, cox regression model demonstrated that serum-based exosomal LINC00917 may act as a potential NSCLC diagnostic biomarker.

While the results of our study are encouraging, drawing a conclusive statement of exosomal LINC00917 being a non-invasive NSCLC biomarker may be premature as certain limitations are associated with our study. First, the numbers of participating NSCLC patients and baseline-matched healthy controls were relatively limited. A multi-Province multi-center cohort study with larger patient population is needed to validate the findings in our study. Second, further clinical investigation, such as examining NSCLC patients’ *in situ* tumors, is needed to specify the origin of exosomal LINC00917 and verify its association with cancer patients’ prognosis. Furthermore, genetic and biochemical approaches are necessary to reveal the possible functional roles of LINC00917 in regulating NSCLC tumor carcinogenesis and development.

## Data Availability

The raw data supporting the conclusion of this article will be made available by the authors, without undue reservation.
